# Neuroprotective effects of VCP modulators in mouse models of glaucoma

**DOI:** 10.1016/j.heliyon.2016.e00096

**Published:** 2016-04-19

**Authors:** Noriko Nakano, Hanako Ohashi Ikeda, Tomoko Hasegawa, Yuki Muraoka, Sachiko Iwai, Tatsuaki Tsuruyama, Masaki Nakano, Tomohiro Fuchigami, Toshiyuki Shudo, Akira Kakizuka, Nagahisa Yoshimura

**Affiliations:** a Department of Ophthalmology and Visual Sciences, Kyoto University Graduate School of Medicine, Kyoto, 606-8507, Japan; b Department of Experimental Therapeutics, Institute for Advancement of Clinical and Translational Science, Kyoto University Hospital, 606-8507, Kyoto, Japan; c Center for Anatomical Studies, Kyoto University Graduate School of Medicine, Kyoto, 606-8507, Japan; d Laboratory of Functional Biology, Kyoto University Graduate School of Biostudies and Solution Oriented Research for Science and Technology, Kyoto, 606-8501, Japan; e Daito Chemix, Ishibashi-cho Fukui-city Fukui 910-3137, Japan

**Keywords:** Cell biology, Neuroscience

## Abstract

Glaucoma is a major cause of adult blindness due to gradual death of retinal ganglion cells. Currently, no therapeutics are available for the protection of these cells from the cell death. We have recently succeeded in synthesizing novel compounds, KUSs (Kyoto University Substances), which can reduce cellular ATP consumption by specifically inhibiting the ATPase activities of VCP, a major ATPase in the cell, and we have shown that KUSs could mitigate the disease progression of rd10, a mouse model of retinitis pigmentosa, without any apparent side effects. Here we show that KUSs (e.g. KUS121 and KUS187) can prevent antimycin- and oligomycin-induced ATP depletion, endoplasmic reticulum (ER) stress, and cell death in neuronally differentiated PC12 cells. Furthermore, KUSs manifest significant efficacies on several mouse models of glaucoma. KUS administration prevented or mitigated ER stress and subsequent apoptotic cell death of retinal ganglion cells in an acute injury mouse model of retinal ganglion cell loss, which was induced with *N*-methyl-D-aspartate. In a mouse model of glaucoma with high intraocular pressure, KUSs prevented the typical glaucoma pathologies, i.e. enlargement of optic disc cupping and thinning of the retinal nerve fiber layer. KUSs also preserved visual functions in GLAST knockout mice, a mouse model for chronic retinal ganglion cell loss. We propose “ATP maintenance” via inhibition of ATPase activities of VCP as a promising new neuroprotective strategy for currently incurable eye diseases, such as glaucoma.

## 1 Introduction

Glaucoma, which is caused by the loss of retinal nerve fibers and retinal ganglion cells, is a leading cause of vision loss worldwide. More than 600 million patients are suffering from this disease, and the number is increasing (Quigley and Broman, 2006). Lowering intraocular pressure by drugs and/or surgery is the only evidence-based treatment. However, it is not always effective; even after the success in reducing the intraocular pressure, visual field loss gradually progresses in many patients. Therefore, alternative treatments, for example, neuroprotection, have long been sought (Chang and Goldberg, 2012). However, there have been no such definitive treatments to protect retinal ganglion cells from cell death. In glaucoma, the involvement of endoplasmic reticulum (ER) stress in the death of ganglion cells has been proposed (Anholt and Carbone, 2013; Doh et al., 2010; Shimazawa et al., 2007; Yang et al., 2011). Thus, new drugs or compounds with ER stress-reducing activities would be promising therapeutics for the protection of retinal ganglion cells from glaucoma-causing insults.

We have long been working on the analysis of the molecular bases of neurodegeneration, especially in “polyglutamine diseases” (Ikeda et al., 1996; Kakizuka, 1998; Kawaguchi et al., 1994). We found that VCP (valosin-containing protein) was profoundly involved in polyglutamine-induced neurodegenerative phenotypes (Higashiyama et al., 2002; Hirabayashi et al., 2001). In our *Drosophila* model of polyglutamine disease, a mutation in Ter94 (Drosophila ortholog of VCP) dramatically reduced neuronal degeneration. Consistently, overexpression of wild-type Ter94 together with polyglutamines exacerbated the neurodegeneration. Furthermore, *VCP* has been identified as a causative gene for IBMPFD (Inclusion Body Myopathy with Paget disease of bone and Frontotemporal Dementia) (Watts et al., 2004) and for rare cases of familial ALS (amyotrophic lateral sclerosis) (Johnson et al., 2010). Both diseases show dominant inheritance, and all identified *VCP* gene mutations create single amino acid substitutions. In our analyses, all examined mutated VCP proteins had elevated ATPase activities, and the relative increase in activity levels appeared to be correlated with the severity of the clinical phenotypes (Manno et al., 2010). From these results, we assumed that specific inhibitors of the VCP ATPase activities may ameliorate the disease phenotypes of familial VCP diseases as well as cell death in other neurodegenerative diseases.

Recently, we have succeeded in developing novel VCP modulators, KUSs (Kyoto University Substances), small chemical compounds that were selected from about 200 newly synthesized compounds based on inhibition of the ATPase activity of VCP (Ikeda et al., 2014). We have shown that under various stress conditions in cultured cells, KUSs were able to significantly maintain cellular ATP levels, and consequently suppress ER stress and cell death (Ikeda et al., 2014). In addition, in rd10, a mouse model of retinitis pigmentosa, KUSs were also able to suppress ER stress, protected photoreceptor cell death, and preserve visual functions (Ikeda et al., 2014). It is notable that VCP is highly expressed in all types of retinal neuronal cells (Ikeda et al., 2014), including retinal ganglion cells, which are lost in glaucoma. This motivated us to investigate the neuroprotective efficacy of KUSs on retinal ganglion cells in several mouse models of glaucoma, and our investigations in this study indicate that KUSs represent a new neuroprotective strategy for currently incurable glaucoma.

## 2 Results

### 2.1 KUSs prevent ATP depletion, ER stress, and cell death in PC12 cells treated with inhibitors of the mitochondrial respiratory chain

**Fig. 1:**
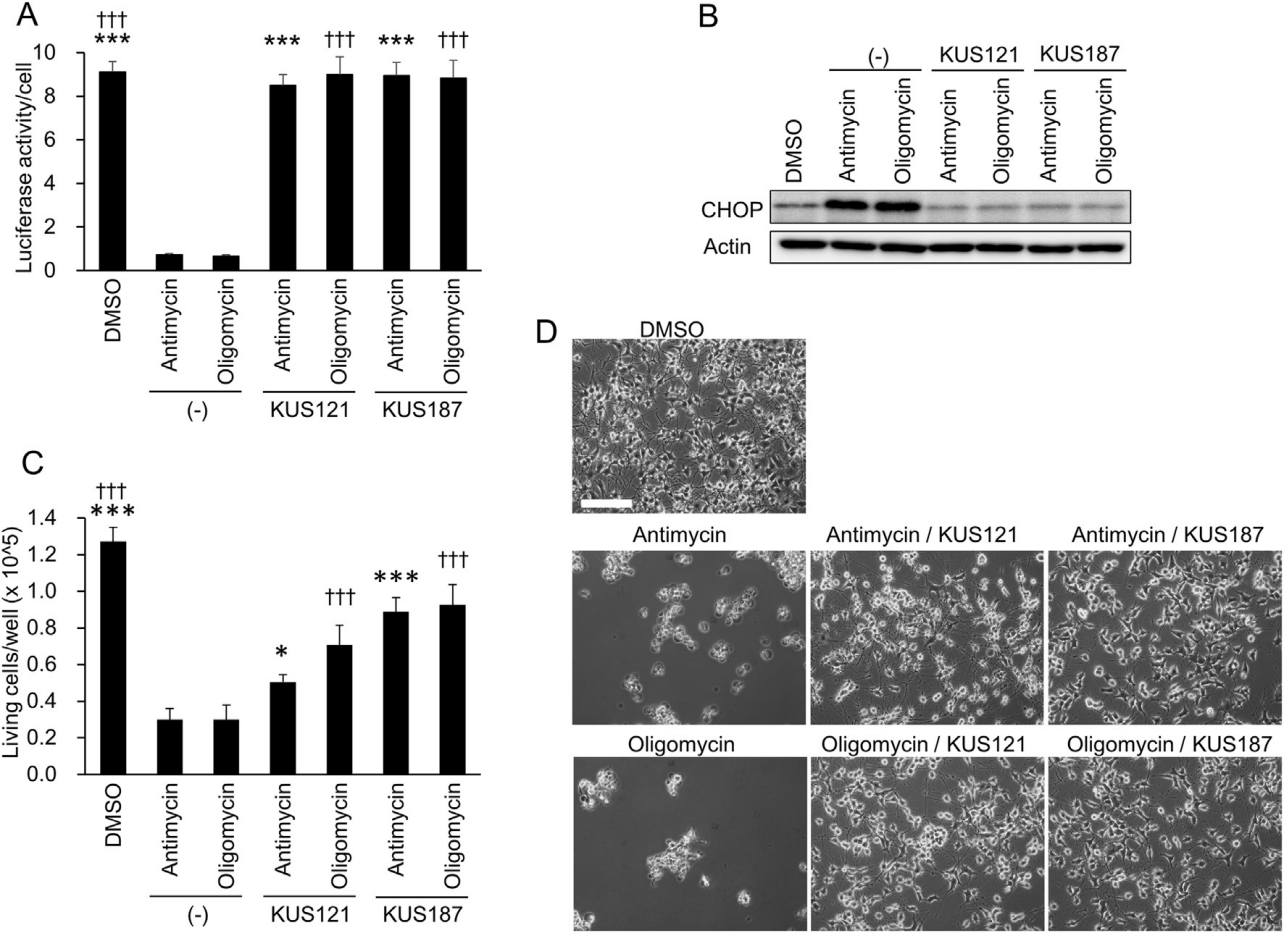
Effects of KUS121 and KUS187 on neuronal PC12 cells treated with mitochondrial respiratory chain inhibitors. (A) ATP levels per cell were measured with luciferase assays. Neuronally differentiated PC12 cells were treated with 100 nM antimycin or 0.01 μg/mL oligomycin for 20 h in the presence of 50 μM KUSs (KUS121 or KUS187) or vehicle (DMSO, −), and were harvested. Total ATP amounts and live cell numbers were measured, and then ATP amounts per cell were calculated (see “Materials and methods”). ***P < 0.005, ^†††^*P* < 0.005, vs. antimycin (*), vs. oligomycin (^†^) (ANOVA with Games-Howell post hoc test). (B) Western blot analyses of CHOP. Neuronally differentiated PC12 cells, cultured in the same conditions as in (A), were harvested, and subjected to western blot analyses. Actin served as a loading control. Complete scans of the different blots are presented in Supplementary Fig. S1. (C, D) Examination of cell viability. Neuronally differentiated PC12 cells were treated with 100 nM antimycin or 0.01 μg/mL oligomycin for 28 h in the presence of 50 μM KUSs (KUS121 or KUS187) or vehicle (DMSO, −). Then, live cell numbers were counted by staining with trypan blue (C) or cells were photographed (D). Mean values of quadruplicate experiments are shown. Bars indicate standard deviations. *P < 0.05, ***P < 0.005, ^†††^*P* < 0.005, vs. antimycin (*), vs. oligomycin (^†^) (ANOVA with Tukey post hoc test). Scale bar: 200 μm.

We first examined the effects of KUSs, namely KUS121 and KUS187, on neuronally differentiated PC12 cells treated with antimycin or oligomycin, which inhibit mitochondrial respiratory chain complex III and V, respectively. As expected, after 20 h of treatment, both inhibitors significantly reduced cellular ATP levels. Under this condition, clear ER stress was evoked, which was evidenced by the induction of C/EBP-homologous protein (CHOP), a well-known ER stress marker (Zinszner et al., 1998). After 28 h of treatment, approximately 80% of the cells were dead (Fig. 1). In contrast to these cells, in cells simultaneously treated with KUSs and these inhibitors, the cellular ATP decrease was significantly inhibited, almost no CHOP induction was observed, and the subsequent cell death was significantly reduced (Fig. 1). These results confirmed our previous results of profound links between a decrease of cellular ATP levels, ER stress, and subsequent cell death, and the abilities of KUS121 and KUS187 to suppress all of them (Ikeda et al., 2014).

### 2.2 KUSs rescue acute retinal ganglion cell injury induced by *N*-methyl-D-aspartate

**Fig. 2:**
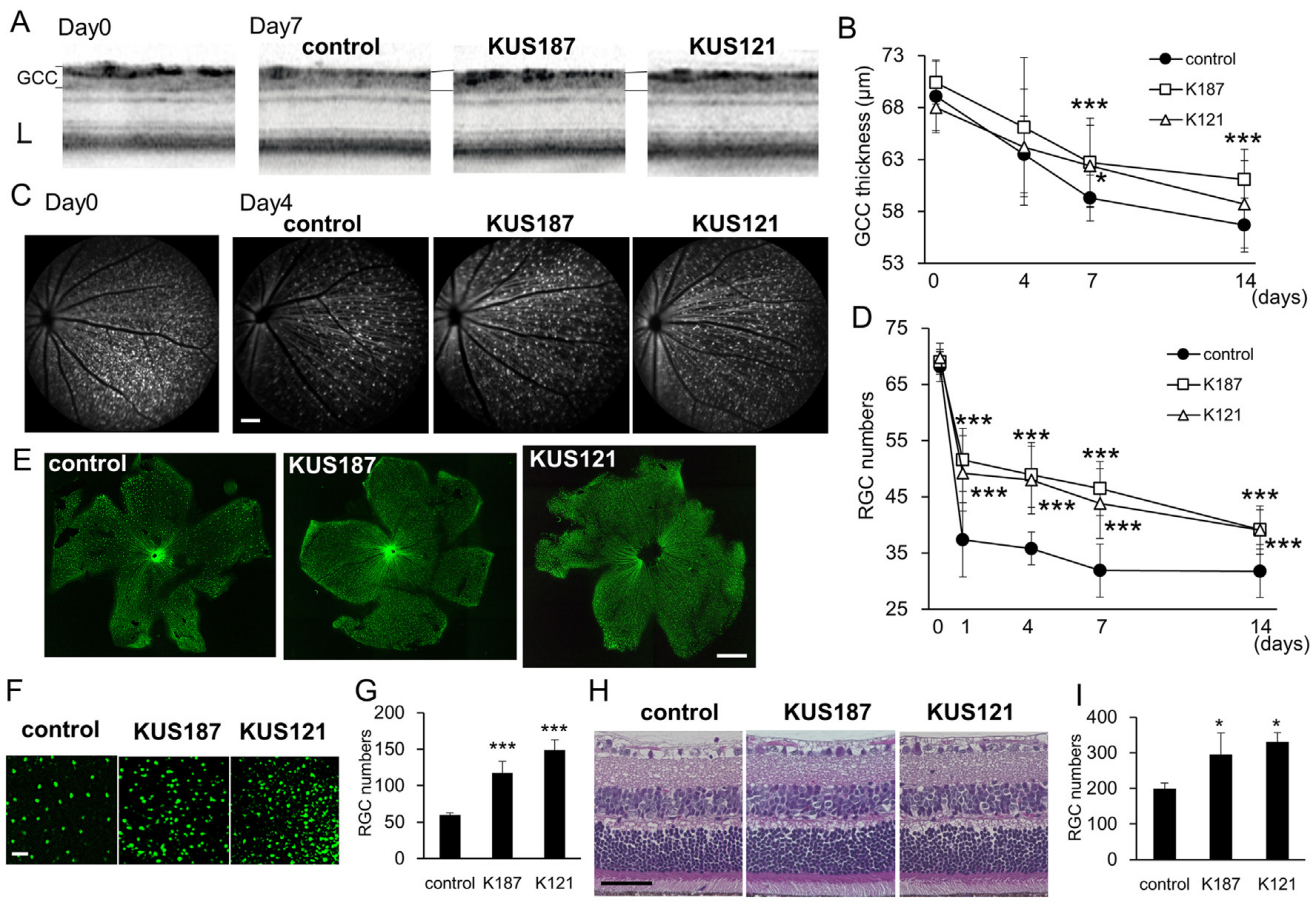
*In vivo* efficacies of KUSs on an acute retinal ganglion cell injury model induced by NMDA. (A) Representative images of spectral domain optical coherence tomography (SD-OCT) taken before (Day 0) or 7 days after NMDA (2 nmol) injection (Day 7) in Thy1-CFP mice that were administered phosphate buffered saline (PBS, control), KUS187, or KUS121. (B) Time-dependent changes of the thickness of retinal ganglion cell (RGC) related inner retinal layers (ganglion cell complex (GCC)), measured by SD-OCT in the mice administered PBS (control, n = 15), KUS187 (K187, n = 17), or KUS121 (K121, n = 26). (C) Representative short-wave-length confocal scanning laser ophthalmoscope (scSLO) images taken before (Day 0) or 4 days after NMDA injection (Day 4). (D) Time-dependent changes in the number of Thy1-CFP-positive RGCs counted in scSLO images. (E, F, G) Analyses on retinal flatmounts from the eyes in Thy1-CFP mice administered PBS (control, n = 5), KUS187 (K187, n = 5), or KUS121 (K121, n = 4) 1 day after NMDA injection. (E) Representative retinal flatmount images. (F) Magnifications of the images in (E). (G) Numbers of RGCs in retinal flatmounts. (H) Hematoxylin and eosin (HE)-stained retinal sections of mice administered PBS (control), KUS187, or KUS121, 1 days after NMDA injection. (I) Numbers of RGCs counted in the HE-stained sections (n = 3). *P < 0.05, **P < 0.01, ***P < 0.005 vs. control (Dunnett’s test) in B, D, G, and I. Scale bars: 40 μm in (A); 100 μm in (C) and (F); 1000 μm in (E) and 50 μm in (H).

We also previously showed that in retinal organ culture KUSs protected retinal ganglion cells from *N*-methyl-D-aspartate (NMDA)-induced cell death (Ikeda et al., 2014). We therefore tested the neuroprotective effects of KUSs on retinal ganglion cells *in vivo*. NMDA was intravitreally injected in Thy1-CFP transgenic mice (Feng et al., 2000) (referred to as Thy1-CFP mice hereafter), in which cyan fluorescent protein (CFP) is expressed in retinal ganglion cells under the Thy1 promoter (Caroni, 1997; Vidal et al., 1990). One week before the NMDA injection, we started to orally administer KUS121 (50 mg/kg/day), KUS187 (50 mg/kg/day), or phosphate buffered saline (PBS) as a control, to the mice. We continued the daily administration of KUSs up to 14 days after the NMDA injection. Spectral-domain optical coherence tomography (SD-OCT) revealed that the thickness of the inner retina (ganglion cell complex (GCC)), which consists of the retinal nerve fiber layer, ganglion cell layer, and inner plexiform layer) (Nakano et al., 2011) gradually decreased in control Thy1-CFP mice. By contrast, the extent of retinal nerve fiber layer thinning in the mice administered KUS121 or KUS187 was milder than the control (Fig. 2A and B). Seven days after NMDA injection, the GCCs of the mice administered KUSs were significantly thicker than those of the control mice (*P* = 0.026 and *P* = 0.012, vs. control, Dunnett’s test, KUS187 and KUS121, respectively, Fig. 2A and B). In control Thy1-CFP mice, CFP-positive retinal ganglion cells rapidly decreased within one day after the NMDA injection, and kept decreasing until day 14 (Fig. 2C and D). For example, 4 days after NMDA injection, the retinal ganglion cell loss was observed even in the group that received KUSs, but this cell loss was attenuated compared with the control group (Fig. 2C). In Thy1-CFP mice administered KUSs, the amount of NMDA-induced retinal ganglion cell death was significantly reduced at each time point compared with the control (Fig. 2D). To confirm the protective effect of KUSs on retinal ganglion cells, retinal flatmounts were analyzed 1 day after the injection. In control retinas, the loss of retinal ganglion cells (Fig. 2E and F) was more severe than in retinas of Thy1-CFP mice administered KUSs, (*P* = 0.002 (KUS121), *P* = 0.003 (KUS187) vs. control, Dunnett’s test, Fig. 2E, F, and G). In addition, retinal nerve fibers were relatively better preserved in the KUS-treated mice than the control mice (Fig. 2E). Preservation of retinal ganglion cells by KUSs was confirmed in retinal sections stained with hematoxylin and eosin (HE) (Fig. 2H and I). These results showed that KUSs administration effectively protected retinal ganglion cells from death induced by NMDA.

### 2.3 KUSs mitigate pathologies of a mouse model of glaucoma with a high intraocular pressure

**Fig. 3:**
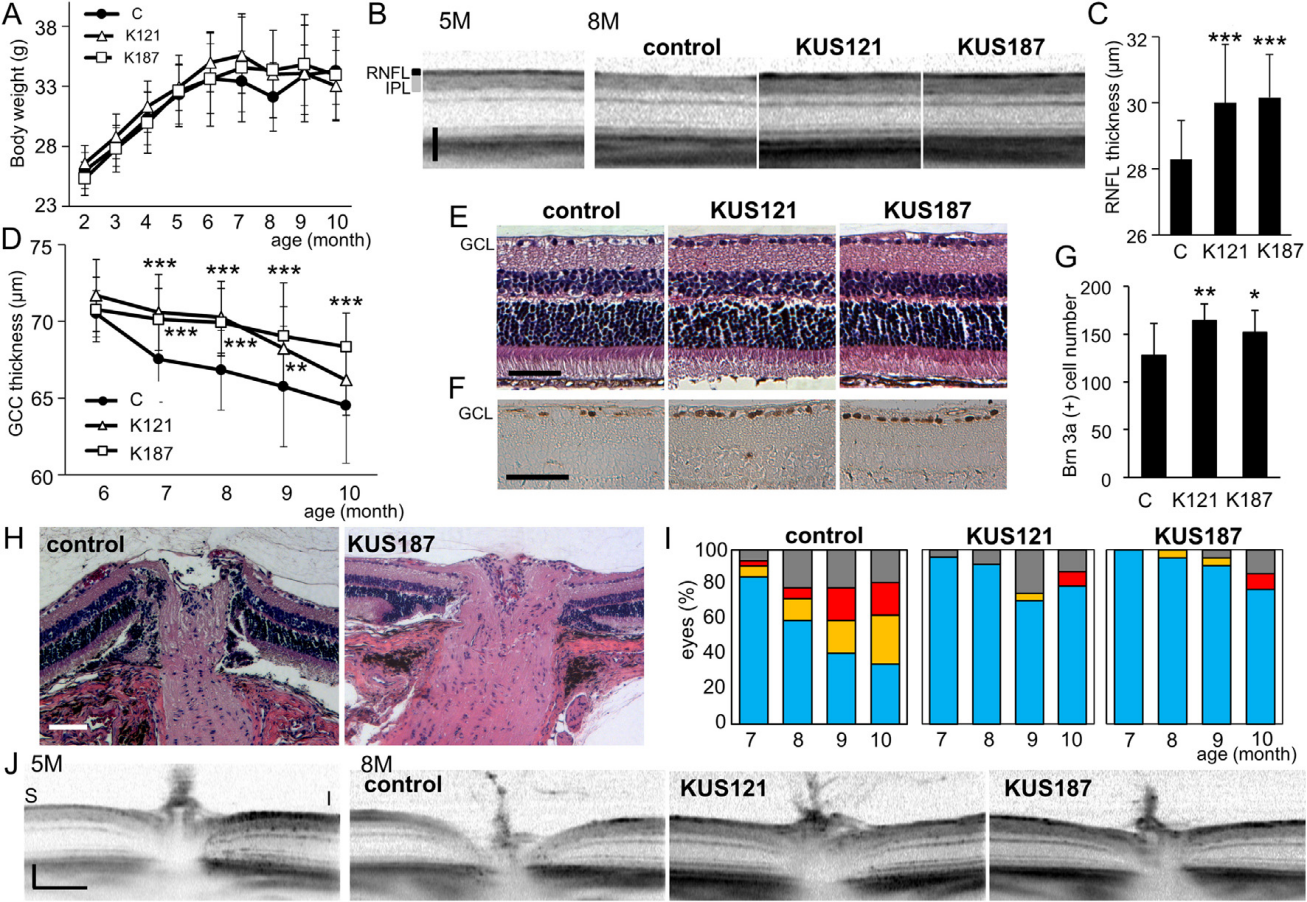
Efficacies of KUSs in DBA/2J mice. (A) Time-dependent changes of body weights in DBA/2J mice administered PBS (control, labeled “C”), KUS121 (K121) or KUS187 (K187). There were no significant differences among the 3 groups of mice in the mean body weight during the experimental periods. (B) Representative images of SD-OCT (vertical scan) of 5-month (5M)- and 8-month (8M)-old DBA/2J mice administered KUS121 (n = 12), KUS187 (n = 11), or PBS as a control (n = 16). The images were taken about 400 μm from the center of the optic nerve head. RNFL: retinal nerve fiber layer. IPL: inner plexiform layer. (C) Comparison of the RNFL thickness in 7-month-old DBA/2J mice administered KUS121 (K121), KUS187 (K187), or PBS (control, labeled “C”). ***P < 0.005, vs. control (Dunnett’s test). (D) Time-dependent changes in thickness of GCC around the optic nerve head of DBA/2J mice, administered KUSs or PBS (control, labeled "C"). **P < 0.005, ***P < 0.001 vs. control (Dunnett’s test). (E, F) HE- (E) or Brn-3a-stained (F) sections of retinas from 10-month-old DBA/2J mice, administered KUSs or PBS. GCL: ganglion cell layer. (G) Comparison of mean numbers of Brn-3a-positive ganglion cells. Fewer retinal ganglion cells were observed in control mice than in KUS-treated mice. **P = 0.010, *P = 0.043, vs. control (labeled "C", Dunnett’s test). (H) Representative images of optic nerve heads (vertical sections) by the HE staining in 10-month-old mice administered PBS or KUS187. (I) Time-dependent changes of cupping of the optic nerve heads observed with SD-OCT. *P* = 0.002, 0.004, KUS121 vs. control, and KUS187 vs. control, respectively (Mann-Whitney U-test). Areas shown in blue, eyes with optic disc cupping less than half of the total retinal thickness; areas in yellow, eyes with optic disc cupping more than half but less than the total retinal thickness; areas in red, eyes with optic disc cupping more than the total retinal thickness; areas in gray boxes, unmeasurable eyes, due to adhesion of pupils etc. (J) Representative images of SD-OCT of the optic nerve heads (vertical sections) from 5- and 8-month-old DBA/2J mice, administered KUS121, KUS187, or PBS as a control. S: superior, I: inferior. Scale bars: 50 μm in (B), (E), and (F) and 100 μm in (H) and (J).

The apparent neuroprotective efficacy of KUSs on retinal ganglion cells *in vivo* prompted us to investigate whether KUSs would have any protective capacity in DBA/2J mice as a model of glaucoma with a high intraocular pressure (Anderson et al., 2002). KUS121 (50 mg/kg/day), KUS187 (50 mg/kg/day), or PBS as a control, were orally administered daily to the mice after the age of 2 months. Mean body weight was monitored throughout the experimental period, and there were no significant differences among KUS- and PBS-administered (control) groups of mice (Fig. 3A). The mean intraocular pressure was similar among the 3 groups. Examinations using SD-OCT monitored time-dependent morphological changes of the retinas in the same mice up to 10 months of age. In aged control DBA/2J mice, the retinal nerve fiber layer (RNFL) became very thin and hard to detect by SD-OCT (Fig. 3B). By contrast, the retinal nerve fiber layer in the KUS-administered DBA/2J mice appeared intact (Fig. 3B). The retinal nerve fiber layer in 7-month-old DBA/2J mice administered KUSs was significantly thicker than in the age-matched control DBA/2J mice (Fig. 3C). Fig. 3D shows time-dependent changes in thickness of the inner retinal layers (GCC). GCC thickness was significantly retained in mice administered KUS121 (e.g., from 7 to 9 months) and KUS187 (e.g., from 7 to 10 months), as compared with control mice. To determine a cut-off value of GCC thickness, we used GCC thickness data of 4 and 5-month-old DBA/2J mice as “normal”, and GCC thickness data of control 10-month-old DBA/2J mice as the standard for glaucoma. When we used 69.50 μm as a cut-off value, the sensitivity and specificity were 0.92 and 0.95, respectively. We estimated retinas with more than 69.5 μm GCC thickness as normal. At the age of 9 months, the percentage of retinas with GCC thicknesses more than 69.5 μm were 15, 29, and 42% in control, KUS121-, or KUS187-treated DBA/2J mice, respectively. Histological examination showed that the majority of retinal ganglion cells, which are positive for Brn-3a, were lost in 10-month-old control DBA/2J mice. By contrast, the ganglion cells and nerve fiber layers in the age-matched KUS-treated DBA/2J mice were preserved (Fig. 3E–G).

We also evaluated optic disc cupping, a characteristic pathology of glaucoma, by histological examination at the age of 10 months (Fig. 3H). Seventy-one percent (5/7 eyes) of the eyes in DBA/2J mice administered PBS showed severe excavation of the optic nerve head. By contrast, in the DBA/2J mice administered KUS121 and KUS187, 7 percent (1/15 eyes) and 10 percent (2/21 eyes) of the eyes, respectively, showed severe excavation. Using SD-OCT, we examined time-dependent morphological changes of the optic nerve heads in the same mice up to 10 months of age. Enlargement of optic disc cupping was observed in many older control DBA/2J mice but only in a few age-matched KUS-administered DBA/2J mice (Fig. 3I and J). With increasing age, the percentage of eyes with enlarged optic disc cupping increased, and the cupping became more severe in the control DBA/2J mice. By contrast, only a small percentage of eyes showed enlargement of the optic disc cupping in the KUS-administered DBA/2J mice (Fig. 3I). These results clearly demonstrate that inhibition of VCP ATPase activities by KUS administration protects ganglion cells *in vivo* in this particular mouse model of high intraocular pressure glaucoma.

### 2.4 KUS mitigates chronic retinal ganglion cell loss in GLAST knockout mice

We next used another glaucoma model, glutamate-aspartate transporter (GLAST) knockout mice (Harada et al., 2007), to confirm the protective effect of KUS on chronic retinal ganglion cell loss. GLAST is the predominant glutamate transporter in the retina. Homozygous (-/-) and heterozygous (+/-) GLAST knockout mice manifest severe and mild gradual retinal ganglion cell loss, respectively, due to the different extents of chronic excitotoxicity caused by excessive glutamate in the retina, which is followed by optic nerve degeneration. These pathologies occur without elevated intraocular pressure (Harada et al., 2007). KUS121 (50 mg/kg/day) or PBS as a control, was intraperitoneally administered after 1 week of age, and orally administered daily after 8 weeks of age.

**Fig. 4:**
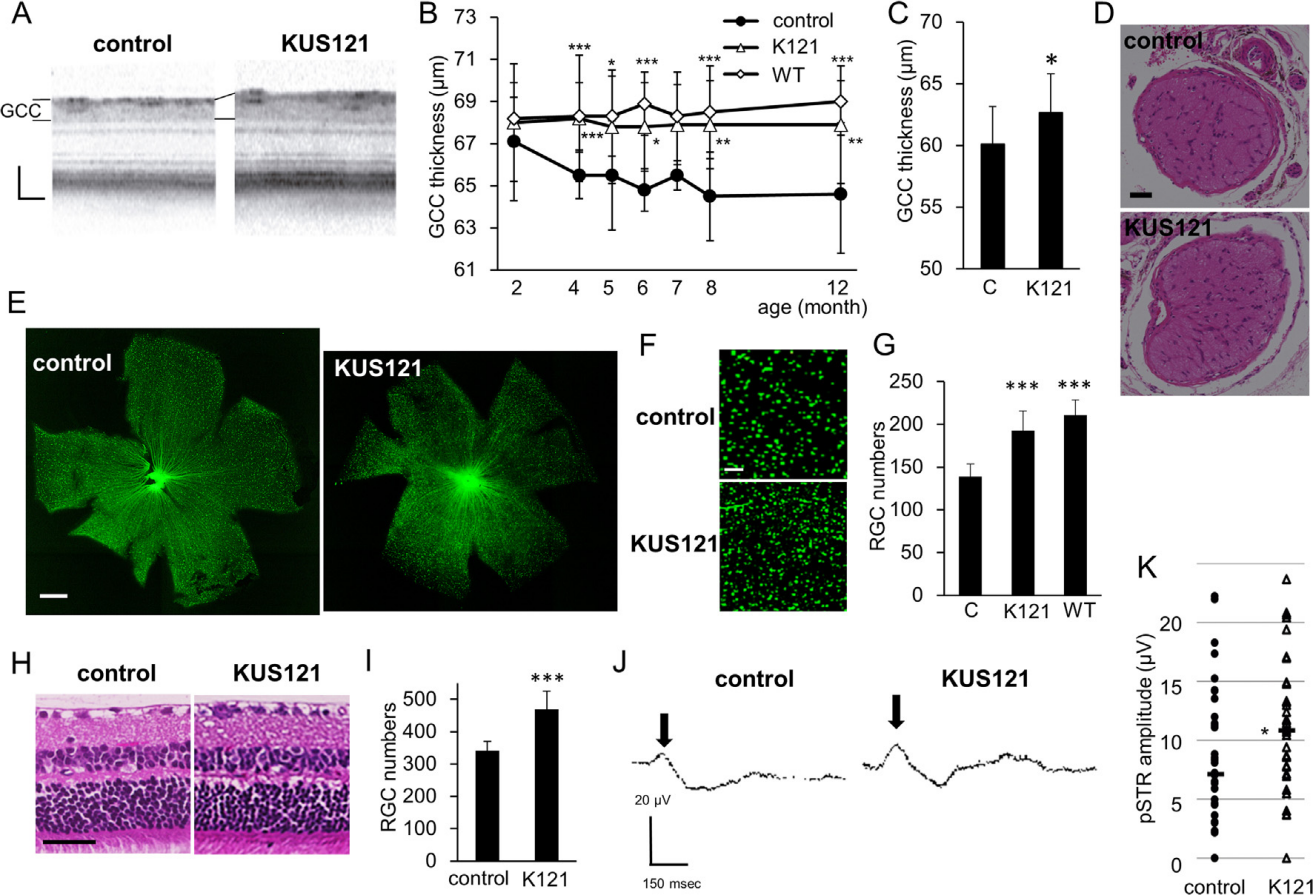
Efficacies of KUS121 on GLAST knockout mice. (A) Representative SD-OCT images, taken at 4 months of age, of GLAST (+/-) mice administered PBS (control) or KUS121. (B) Time-dependent changes of the thickness of GCC measured on the SD-OCT images in GLAST (+/-) mice administered PBS (control, n = 29), KUS121 (K121, n = 44), and wild-type mice (WT, n = 21). *P < 0.01, **P < 0.001, ***P < 0.0001 vs. control (Dunnett’s test). (C) Comparison of thickness of GCC around the optic nerve head of 4-month-old GLAST (-/-) mice administered KUS121 (K121) or PBS (labeled “C”). *P = 0.03 (Student's t-test). (D) Representative images of HE-stained sections of the optic nerve from 4-month-old GLAST (-/-) mice administered KUS121 or PBS (control). (E) Representative retinal flatmount images of the 12-month-old GLAST (+/-) mice administered KUS121 or PBS (control). (F) Magnifications of the images in (E). (G) Number of retinal ganglion cells (RGCs) in retinal flatmounts of GLAST (+/-) mice administered PBS (control, labeled “C”, n = 8), KUS121 (K121, n = 13), and wild-type mice (WT, n = 8). ***P < 0.0001 vs. control (Dunnett’s test). (H) Representative images of HE-stained retinal sections of the 12-month-old GLAST (+/-) mice administered PBS (control) or KUS121. (I) Number of RGCs counted on the HE-stained sections. ***P < 0.0001 vs. control (Aspin-Welch t-test). (J) Representative images of positive threshold response (pSTR) taken on 12-month-old GLAST (+/-) mice administered PBS (control) or KUS121. (K) Comparison of the amplitude of pSTR waves in GLAST (+/-) mice administered PBS (control, n = 36 eyes) or KUS121 (K121, n = 35 eyes). Bar shows the median. *P < 0.05 (Student’s t-test). Scale bars: 40 μm in (A); 50 μm in (D) and (H); 1000 μm in (E) and 100 μm in (F).

SD-OCT examination revealed that the thickness of the inner retina (GCC) gradually decreased between 2 to 12 months of age in the control GLAST (+/-) mice (Fig. 4A and B ). By contrast, the thickness was almost stable in the KUS121-administered GLAST (+/-) mice (Fig. 4A and B). The GCC of the mice administered KUS121 was significantly thicker than that of the control mice at 4, 6, 8 and 12 months of age (Fig. 4B). The preservation of the inner retina and optic nerve was confirmed in 4-month-old GLAST (-/-) mice administered KUS121 (Fig. 4C and D). On retinal flatmounts of the 12-month-old control GLAST (+/-) mice, loss of the retinal ganglion cells and retinal nerve fibers was apparent (Fig. 4E and F). By contrast, loss of the retinal ganglion cells and retinal nerve fibers was mild in KUS121-administered age-matched GLAST (+/-) mice (Fig. 4E and F). The number of remaining retinal ganglion cells in the KUS-administered GLAST (+/-) retinas was significantly greater than in the control GLAST (+/-) mice (*P* < 0.0001 vs. control, Dunnett’s test, Fig. 4G). Preservation of the retinal ganglion cells and retinal nerve fibers in the KUS-administered retina was confirmed in HE-stained retinal sections of 12-month-old mice administered KUS or PBS for 10 months (Fig. 4H and I).

In order to test the function of the remaining retinal ganglion cells, we performed electroretinograms on 12-month-old GLAST (+/-) mice, and measured positive scotopic threshold response (pSTR), which has been shown to originate from the functional inner retina in mice (Saszik et al., 2002). Normal pSTR is observed as the first small positive wave which occurs after a very weak flash light stimulus (Saszik et al., 2002). The amplitudes of the pSTR waves were significantly greater in the KUS121-administered GLAST (+/-) mice than in control GLAST (+/-) mice (Fig. 4J and K). These results demonstrate that KUS121 protected retinal ganglion cells both morphologically and functionally *in vivo* in the mouse model of normal tension glaucoma.

### 2.5 KUS prevent ER stress and apoptotic cell death in glaucoma mouse models

**Fig. 5:**
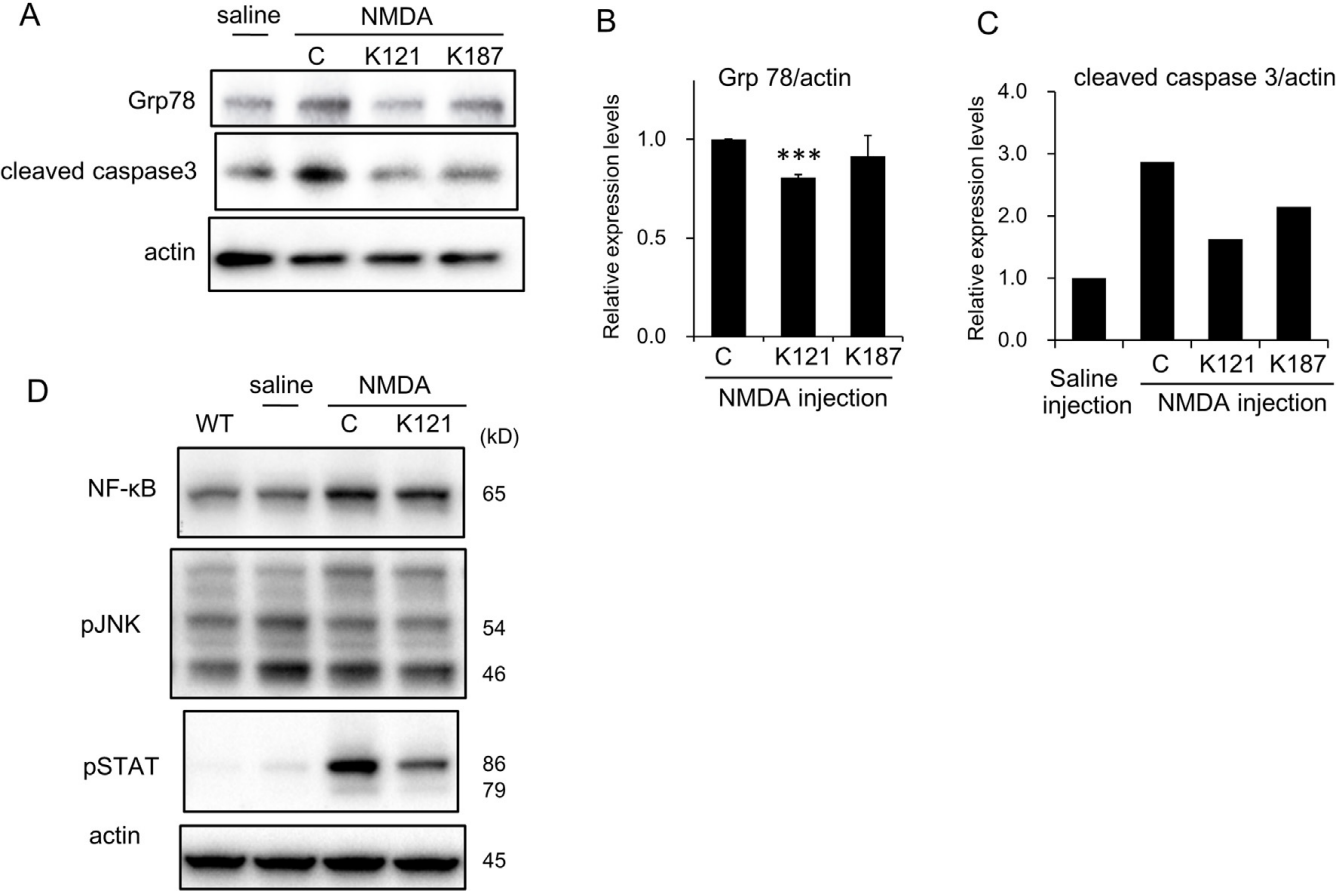
Western blot analyses of KUS-treated retinas from an acute retinal ganglion cell injury model. (A–C) Western blot analyses of retinas 1 day after NMDA injection. (A) The left lane shows signals from a sample of saline-injected mice, and the remaining three lanes show signals from samples of NMDA (5 nmol)-injected mice, with PBS (lane labeled “C”) or KUS (K121 or K187) treatment. Each sample consisted of pooled extracts from 3 retinas, and actin served as a loading control. Complete scans of the different blots are presented in Supplementary Fig. S2. (B, C) Expression levels of Grp78 (B) and cleaved caspase 3 (C) were quantified and relative values are shown. In (B), mean values of three experiments with standard deviations are shown. ***P = 0.004, vs. control (ANOVA with Games-Howell post hoc test). (D) Western blot analyses of retinas 1 day after NMDA injection. The left lane shows signals from a sample of non-treated wild-type mice (WT); the second lane shows signals from a sample of saline-injected mice; and the remaining two lanes show signals from samples of NMDA (5 nmol)-injected mice, with PBS (lane labeled “C”) or KUS121 (K121) treatment. Each sample consisted of pooled extracts from 3 retinas, and actin served as a loading control. Complete scans of the different blots are presented in Supplementary Fig. S3.

**Fig. 6:**
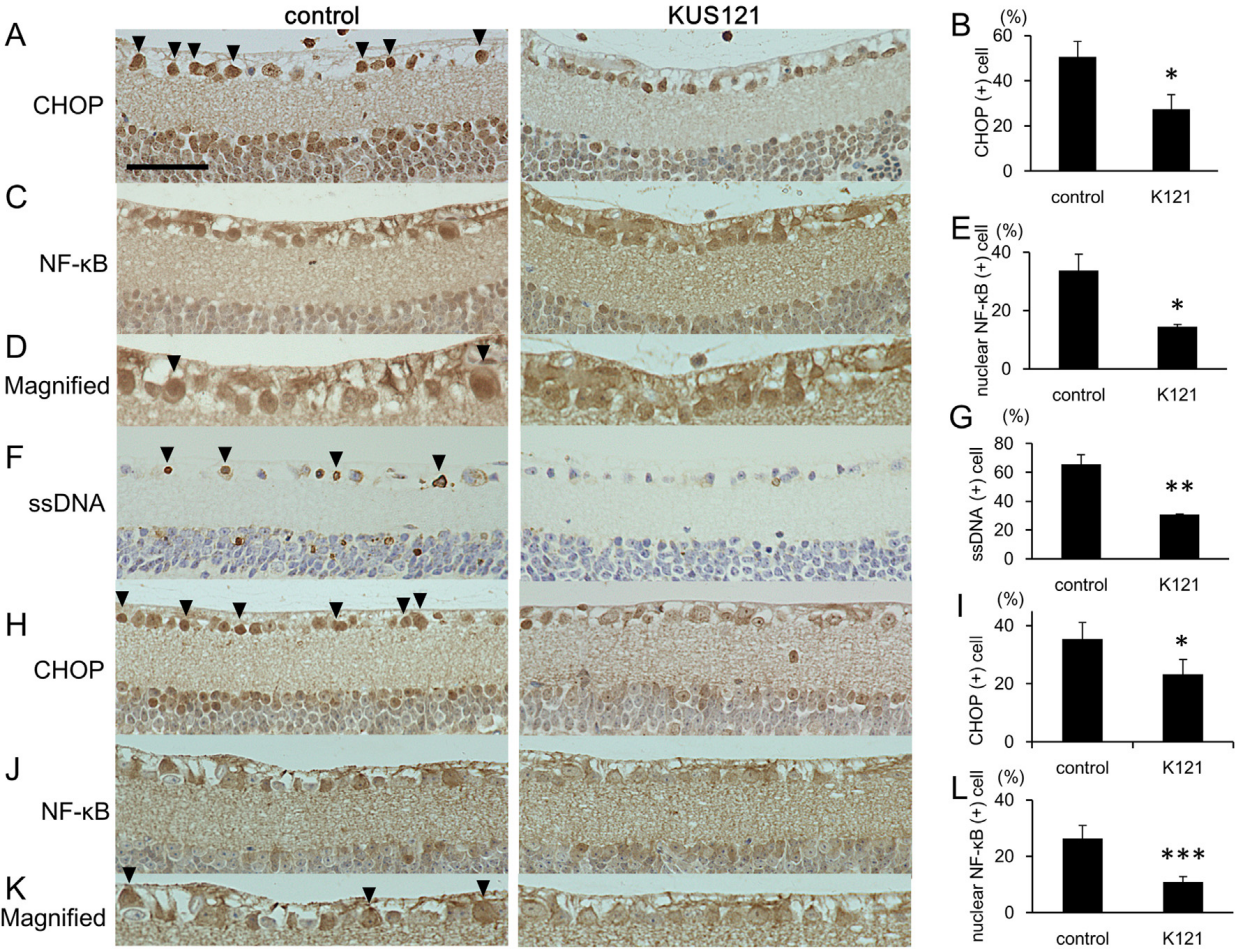
KUS reduces ER stress and prevents apoptotic cell death of retinal ganglion cells in mouse glaucoma models. (A–G) Immunohistochemical analyses of retinas of mice administered PBS (control) or KUS121, 1 day after intravitreal injection of 5 nmol NMDA, interrogated with anti-CHOP (A, B), anti-NF-κB (C, D, E), and anti-single-strand DNA (ssDNA, F, G) antibodies. Arrowheads indicate CHOP- (A), nuclear translocated NF-κB- (D), and ssDNA-positive (F) retinal ganglion cells. (B, E, G) Quantification of CHOP- (B), nuclear translocated NF-κB- (E), and ssDNA-positive (G) retinal ganglion cells in NMDA-injected mice administered PBS (control, n = 3) or KUS121 (K121, n = 3). (H-L) Immunohistochemical analyses of 12-month-old GLAST (+/-) mice with anti-CHOP (H, I) and anti-NF-κB (J, K, L) antibodies. Arrowheads indicate CHOP- (H) and NF-κB-positive (K) retinal ganglion cells. (I, L) Quantification of CHOP- (I) and nuclear NF-κB-positive (L) cells in GLAST (+/-) mice administered PBS (control, n = 4 (NF-κB, CHOP)) or KUS121 (K121, n = 5 (NF-κB), n = 4 (CHOP)). *P < 0.05, **P < 0.001, ***P < 0.0001 (Aspin-Welch t-test). Scale bar: 50 μm.

We have demonstrated that KUSs prevented ER stress in several different conditions both in cultured cells and in mice (Ikeda et al., 2014). Therefore, we tested the effect of prophylactic administration of KUSs on the expression levels of ER stress marker proteins in retinas from mice that received intravitreal injections of NMDA. The expression level of 78 kDa glucose-regulating protein (Grp78, also known as HSPA5 or Bip), an ER stress marker (Kim et al., 2006), was elevated in control NMDA-injected retinas (Fig. 5A). By contrast, induction of Grp78 was suppressed in retinas from mice that had been prophylactically administered KUS121 or KUS187 (Fig. 5A and B). We also performed immunohistochemical staining to examine the expression levels of CHOP, which is a core mediator of ER stress-induced cell death and is upregulated during ER stress (Zinszner et al., 1998). There were many CHOP-positive retinal ganglion cells in control NMDA-injected retinas, and the number of CHOP-positive cells was significantly lower in retinas of KUS121-administered mice than in those of the control mice (Fig. 6A and B). NF-κB is a transcription factor that regulates gene expression in many processes such as the inflammatory response, apoptosis, and ER stress (Jiang et al., 2003; Pahl and Baeuerle, 1995). After NMDA injection, we observed the increased expression of NF-κB (Fig. 5D). The increase of NF-κB expression was not apparently affected by KUS121-treatment. NMDA has been reported to activate JNK (c-Jun N-terminal kinase), followed by the nuclear translocation of NF-κB, which induces apoptotic cell death (Ko et al., 1998; Lam et al., 1999). However, we could not observe any apparent changes of phosphorylation levels of JNK in the retina after NMDA injection, or by KUS121 administration (Fig. 5D). However, we found a significant reduction in nuclear NF-κB in KUS121-treated retinas, compared to those of the control NMDA-injected mice (Fig. 6C–E). Phosphorylation of STAT 3 (Signal Transducer and Activator of Transcription) was induced in the NMDA-injected retinas, and it was suppressed in the KUS121-treated retinas (Fig. 5D). The level of cleaved caspase 3, which is a key executioner protease of apoptosis (Boatright and Salvesen, 2003), increased in control retinas injected with NMDA. The NMDA-induced increase in cleaved caspase 3 was suppressed in retinas from KUS-administered groups (Fig. 5A and C). To identify apoptotic cells, we stained single-stranded DNA (ssDNA). The percentage of ssDNA-positive cells was significantly lower in retinas of KUS121-administered, NMDA-injected mice than in those of the control NMDA-injected mice (Fig. 6F and G).

Consistent with a role of KUSs in the suppression of ER stress, immunohistochemical examination of retinas from 12-month-old GLAST (+/-) mice revealed significantly fewer CHOP-positive (ER-stress-positive) retinal ganglion cells in KUS121-administered GLAST (+/-) mice than in the age-matched control GLAST (+/-) mice (Fig. 6H and I). Likewise, retinal ganglion cells with nuclear NF-κB were significantly fewer in KUS121-administered GLAST (+/-) mice than in age-matched control GLAST (+/-) mice (Fig. 6J–L). These results, as a whole, provide strong evidence that KUSs are able to suppress ER stress and to prevent apoptotic cell death of retinal ganglion cells in response to different insults.

## 3 Discussion

In this study, we provided several lines of evidence that KUSs, which we previously developed as specific inhibitors of the ATPase activities of VCP (Ikeda et al., 2014), have neuroprotective effects on both cultured PC12 cells and mouse ganglion cells *in vivo*. More precisely, KUSs were able to protect neuronally differentiated PC12 cells from cell death induced by the treatment of antimycin and oligomycin. Antimycin and oligomycin inhibit mitochondrial respiratory chain complex III and V, respectively, and thus lead to decreases in ATP levels and subsequent cell death. Furthermore, we showed that KUSs significantly retarded morphological and functional progressions of the glaucoma phenotypes in several different mouse models of glaucoma.

In glaucoma, several etiologies have been proposed, including glutamate exitotoxicity (Doucette et al., 2015; Dreyer et al., 1996; Siliprandi et al., 1992), mitochondrial dysfunction (Chrysostomou et al., 2013), ER stress (Doh et al., 2010), etc., for the mechanism of retinal ganglion cell death. These etiologies are likely not mutually exclusive but rather intertwined events. For example, glutamate excitotoxicity has been proposed to induce mitochondrial membrane depolarization or dysfunction (Ruiz et al., 2009). Mitochondrial dysfunctions have also been suggested in other glaucoma models, e.g. DBA/2J (Coughlin et al., 2015), in which a high intraocular pressure has been shown to cause the retinal decrease of ATP (Baltan et al., 2010). In addition, there are many reports showing that NMDA and high intraocular pressure induce ER stress, which leads to neuronal cell death (Awai et al., 2006; Concannon et al., 2008; Doh et al., 2010; Ruiz et al., 2009; Shimazawa et al., 2007). We showed that KUS administration reduced Grp78 or CHOP expression and retinal ganglion cell death in three different mouse glaucoma models (Fig. 5 and Fig. 6). These data are consistent with our analyses of neuronally differentiated PC12 cells (Fig. 1) as well as our previous data (Ikeda et al., 2014), both demonstrating that KUSs are able to suppress ER stress and subsequent cell death.

In neuronally differentiated PC12 cells, VCP is apparently responsible for up to about 40% of the total soluble cellular ATPase activities (Ikeda et al., 2014), and thus the suppression of VCP-specific ATPase activity by KUSs would presumably diminish total cellular ATP consumption substantially. This large VCP-specific contribution to cellular ATP consumption might also generally occur in neuronal cells, because terminally differentiated neuronal cells no longer utilize ATP for cell division, a major ATP demand in dividing cells. The binding of ER stress sensors, e.g. PKR-like endoplasmic reticulum kinase (Perk) and inositol-requiring enzyme-1 (Ire1), to Grp78 (Bip) has been shown to be ATP-dependent (Sou et al., 2012). Thus, in a possible scenario, a decrease in the ATP level in the cell or eventually in the ER induces the dissociation of Bip from the ER-stress sensors, leading to their self-oligomerization and subsequent activation. This scenario is consistent with our data demonstrating that KUSs significantly prevented ATP depletion in antimycin- or oligomycin-treated PC12 cells, inhibited CHOP expression, a well-known ER stress marker, and prevented cell death (Fig. 1).

In certain cases of ER stress, but not always, it has been reported that ER stress induces NF-κB activation without affecting JNK activation, in which Ire1 functions as a major mediator of IκB degradation (Tam et al., 2012). Similar events might occur in the NMDA-induced acute glaucoma model as well as in GLAST (+/-) mice. In these models, control mice showed significant increases of CHOP-expressing ganglion cells and ganglion cells with nuclear NF-κB (Fig. 6). In these retinas, we could not observe an increase in phosphorylated JNK (Fig. 5). In KUS-treated retinas of these mouse models, the numbers of ganglion cells expressing CHOP or nuclear NF-κB were significantly suppressed (Fig. 6). Furthermore, our observation of reduced CHOP expression, less cleaved caspase 3 (Fig. 5), and fewer ssDNA-positive retinal ganglion cells in KUS-treated retinas (Fig. 6) clearly demonstrated that KUS-treatment diminished ER stress and subsequent apoptotic cell death.

As a transcription factor, nuclearly translocated NF-κB generally elicits an inflammatory cascade or produces inflammatory cytokines, which in turn activate STAT 3 via their binding to the respective receptors. Indeed, we observed concomitant increases in abundance of nuclear NF-κB and activated STAT 3 in NMDA-injected retinas, and both were suppressed by the KUS administration (Fig. 5 and Fig. 6). Roles of STAT 3 in response to several stresses have been examined (Nicolas et al., 2013). It has been reported that sustained activation of STAT 3 by phosphorylation plays an important role in neuroprotection of NMDA-induced retinal ganglion cell injury (Zhang et al., 2008). On the other hand, others reported that activation of STAT 3 increases apoptosis (Dedoni et al., 2010). Although our results are consonant with the idea that both NF-κB and STAT 3 contribute to neurotoxicity, precise roles of STAT 3 and NF-κB, either for neurotoxic or neuroprotective functions, in these glaucoma models remain to be clarified.

In conclusion, KUSs could provide convenient novel therapeutics for neuroprotection in incurable eye diseases such as glaucoma. Given that the major pathology of many incurable human disorders, e.g. neurodegenerative diseases, ischemic diseases, etc., is early cell death in the affected organs, which occurs long before somatic death, KUSs could also provide a novel strategy for cell protection in these disorders.

## 4 Materials and methods

### 4.1 Cell culture

PC12 cells were cultured with low glucose D-MEM (Nacalai Tesque, Kyoto, Japan), supplemented with 10% FBS (Sigma) and 5% horse serum (Sigma). 7.5 × 10^4^ PC12 cells were treated with 50 ng/mL NGF (Alomone labs, Jerusalem, Israel) for 24 h, then antimycin (Sigma), oligomycin (Sigma), and KUSs were added as indicated in Results, and cells were cultured for another 20 or 28 h. ATP in cultured cells was measured with an ARVO multilabel counter, using a luciferase-based ATP assay reagent for cells (Toyo B-net, Tokyo, Japan). For western blotting, 15 μg whole cell extracts were loaded per lane.

### 4.2 Animal experiments

All studies were conducted in compliance with the ARVO Statement for the Use of Animals in Ophthalmic and Vision Research. All protocols were approved by the Institutional Review Board of Kyoto University Graduate School of Medicine (MedKyo11229, 12245, 13221, 14213). Transgenic mice, B6.Cg-Tg (Thy1-CFP) 23Jrs/J (referred to as ‘Thy1-CFP mice’ in this study) (Feng et al., 2000) were obtained from the Jackson Laboratory (Bar Harbor, ME, USA) and DBA/2J mice (Anderson et al., 2002) from CLEA (Shiga, Japan). GLAST knockout mice (Harada et al., 2007) were a gift from Dr. Koichi Tanaka (Tokyo medical and dental university). GLAST knockout mice and Thy1-CFP mice were crossbred to make GLAST (+/-): Thy1-CFP mice. Genotyping was performed by PCR as recommended by Jackson Laboratory and by Dr. K Tanaka. The environment was maintained at a 14-h light/10-h dark cycle. All mice were fed ad libitum. Male mice (age, 8 weeks; weight, 25–30 g) were used in the experiments. Before image or electroretinography acquisition, mice were anesthetized with an intramuscular injection of a ketamine (70 mg/kg)/xylazine (14 mg/kg) mixture, or pentobarbital (50 mg/kg) for the NMDA-injected model. Pupils were dilated to a diameter of approximately 2 mm with tropicamide and phenylephrine (0.5% each) eye drops.

Two or 5 nmol of NMDA was intravitreally injected with a 33-gauge needle in Thy1-CFP mice to make an acute retinal ganglion cell injury model.

### 4.3 Administration of KUSs

KUS121 or KUS187 was dissolved in 5% Cremophor EL (Sigma) in phosphate buffered saline (PBS) to make a 5 mg/mL solution. In the NMDA-injected mouse model, daily oral administration of KUS121 or KUS187 (Ikeda et al., 2014) (50 mg/kg, 10 mL/kg) was started 1 week before NMDA injection. In DBA/2J mice, KUS121 or KUS187 was orally administered starting at 2 months of age. In GLAST heterozygous or homozygous mice, KUS121 (50 mg/kg) was intraperitoneally administered after 1 week, and orally administered daily after 8 weeks. 5% Cremophor/PBS without KUSs (PBS) was administered as a control. Intraperitoneal injection of 50 mg/kg of KUS121 or KUS187 showed cell protection in a mouse model for retinitis pigmentosa (Ikeda et al., 2014). In pilot experiments using several mouse models, oral administration of 50 mg/kg of KUS121 or KUS187 showed neuroprotective effects. Therefore, we chose oral administration of at least 50 mg/kg of KUS121 or KUS187 in these experiments. Because oral administration in mice younger than 2 months was difficult, intraperitoneal injection was used in young GLAST (+/-) or (-/-) mice.

### 4.4 SD-OCT and scSLO examination

Spectral-domain optical coherence tomography (SD-OCT) examinations using *Multiline OCT* (Heidelberg Engineering, Heidelberg, Germany) were performed (Nakano et al., 2011) in NMDA-injected mice at day 0, 4, 7, and 14; DBA/2J mice monthly beginning at the age of 6 months; and GLAST (+/-): Thy1-CFP mice at 2, 4, 5, 6, 7, 8, and 12 months of age. The thickness of the inner retina (GCC, which includes retinal nerve fiber layer, retinal ganglion cell layer, and inner plexiform layer) around the optic nerve head was measured using circle scan images (Nakano et al., 2011). Optic disc cupping in DBA/2J mice was evaluated using SD-OCT images of vertical scans through the optic nerve head. In NMDA-injected mice, scSLO imaging was simultaneously performed with SD-OCT imaging. For retinal ganglion cell imaging with scSLO, CFP-positive retinal ganglion cells were manually counted within 4 square 310 × 310 μm areas at a distance of 830 μm from the center of the optic nerve head on the scSLO images in a masked fashion. The numbers of counted retinal ganglion cells from the 4 square areas were averaged.

### 4.5 Electroretinography

Electroretinography was performed in GLAST (+/-) mice at 12 months of age. Mice were dark-adapted overnight before anesthetization. Positive threshold response (pSTR) (Saszik et al., 2002) was recorded using a gold loop corneal electrode with a light-emitting diode (Mayo Corp., Inazawa, Japan). A reference electrode was placed in the mouth and a ground electrode was inserted into the anus. Stimuli were produced with a light emitting diode stimulator (3.98 cd m^−2^, for 10 μsec, -4.40 log cd s m^−2^, Mayo Corp.). Up to 100 responses were averaged to obtain the final pSTR (PowerLab 2/25; AD instruments, New South Wales, Australia).

### 4.6 Histological evaluation of retinas

Eyeballs of NMDA-injected mice at day 1, 10-month-old DBA/2J mice and 12-month-old GLAST (+/-) mice were enucleated after pentobarbital overdose. A suture was placed on the edge of the superior conjunctiva to identify the superior portion of the retina. The eyes were fixed in 4% paraformaldehyde for 24 h at 4 °C and embedded in paraffin. Serial 6-μm paraffin-embedded sections were cut through the suture and at the point of insertion of the optic nerve. The sections that passed through the center of the optic nerve head were selected. The selected retinal sections were stained with HE or with each antibody, and photographed about 400 μm (for NMDA-injected mice and DBA/2J mice) or 600 μm (for GLAST knockout mice) apart from the center of the optic nerve head under an optical microscope (Axioplan 2; Carl Zeiss Jena GmbH, Jena, Germany, BZ-9000, Keyence). NF-κB p65 nuclear translocated cells, CHOP-positive, or Brn-3a-positive cells in the ganglion cell layer were counted in vertical paraffin sections through the center of the optic nerve head.

### 4.7 Antibodies

Anti-Grp78, anti-NF-κB, anti-cleaved caspase 3 (Asp175), anti-phospho-JNK (Thr183/Tyr185) and anti-phospho STAT 3 (Tyr705) antibodies were purchased from Cell Signaling (MA,USA); anti-actin and anti-Brn-3a from Chemicon (MA, USA); anti-CHOP from Santa Cruz Biotechnology (CA, USA); anti-ssDNA from Immuno-Biological Laboratories (Fujioka, JAPAN).

### 4.8 Retinal flatmounts

After 1 h fixation in 4% paraformaldehyde, retinal flatmounts without sclera and uvea were made. In the retinal flatmounts, retinal ganglion cells were manually counted within 4 square 700 × 700 μm areas at a distance of 1700 μm from the center of the optic nerve head.

### 4.9 Statistical analysis

For statistical analyses in experiments using PC12 cells and western blot analyses of retinal samples, ANOVA with Games-Howell (if variances were unequal, comparison of luciferase activity) or Tukey (if variances were not unequal, comparison of living cell numbers) post hoc test was used. Variables among mice administered or without KUSs were compared with the following statistical tests: Dunnett’s test (experiments on NMDA-induced acute retinal ganglion cell injury model mice, comparison of RNFL and GCC thickness in DBA/2J mice and GLAST (+/-) mice, comparison of retinal ganglion cell numbers in DBA/2J mice and in retinal flatmounts of GLAST (+/-) mice); Mann-Whitney U-test (comparison of severity of optic nerve cupping); Student’s t-test (if variances were not unequal, comparison of GCC thickness in GLAST (-/-) mice and pSTR amplitudes in GLAST (+/-) mice), or Aspin-Welch t-test (if variances were unequal, comparison of retinal ganglion cell numbers on HE-stained sections of GLAST (+/-) mice immunohistochemical analyses). Statistical analysis was performed using PASW Statistics version 17.0 (SPSS Inc., Chicago, IL). The level of statistical significance was set at *P* < 0.05.

## Declarations

### Author contribution statement

Noriko Nakano, Hanako O. Ikeda: Conceived and designed the experiments; Performed the experiments; Analyzed and interpreted the data; Wrote the paper.

Tomoko Hasegawa, Yuki Muraoka, Sachiko Iwai, Tatsuaki Tsuruyama, Masaki Nakano: Performed the experiments.

Tomohiro Fuchigami, Toshiyuki Shudo: Contributed reagents, materials, analysis tools or data.

Akira Kakizuka: Wrote the paper.

Nagahisa Yoshimura: Conceived and designed the experiments; Contributed reagents, materials, analysis tools or data.

### Funding statement

This research was supported in part by Research grants from the Astellas Foundation for Research on Metabolic Disorders, the Japan Foundation for Applied Enzymology, the Uehara Memorial Foundation, Mochida Memorial Foundation for Medical and Pharmaceutical Research, YOKOYAMA Foundation for Clinical Pharmacology (YRY1308), Japan Intractable Diseases Research Foundation, Japan Research Foundation for Clinical Pharmacology, ONO Medical Research Foundation, Takeda Science Foundation, Japan National Society for the Prevention of Blindness, a Grant-in-Aid for Young Scientists (24791850, H.O.I), grants from SORST of JST (A.K.), the Ministry of Education, Culture, Sports, Science, and Technology of Japan (A.K., H.O.I. and N.Y.), and the Ministry of Health, Labour and Welfare of Japan (A.K., H.O.I. and N.Y.) and the Innovative Techno-Hub for Integrated Medical Bio-Imaging of the Project for Developing Innovation Systems (N.Y.) from the Ministry of Education, Culture, Sports, Science, and Technology of Japan.

### Competing interest statement

The authors declare the following conflict of interests: in relation to this manuscript, Kyoto University and Daito Chemix applied for patents (PCT/JP2011/067320 & PCT/JP2011/073160), and H.O.I., N.N., T.F., T.S., N.Y. & A.K. were inventors of the applied patents. The other authors declare no competing interests.

### Additional information

Supplementary content related to this article has been published online at 10.1016/j.heliyon.2016.e00096.

No additional information is available for this paper.
