# Supplementary material for: Neuroprotective effects of VCP modulators in mouse models of glaucoma
Source: Heliyon. 2016 Apr 19;2(4):e00096. doi: 10.1016/j.heliyon.2016.e00096 (PMC4946081; doi:10.1016/j.heliyon.2016.e00096)
Supplement: Supplementary Figures [file mmc1.pdf]

Supplementary Figure S1

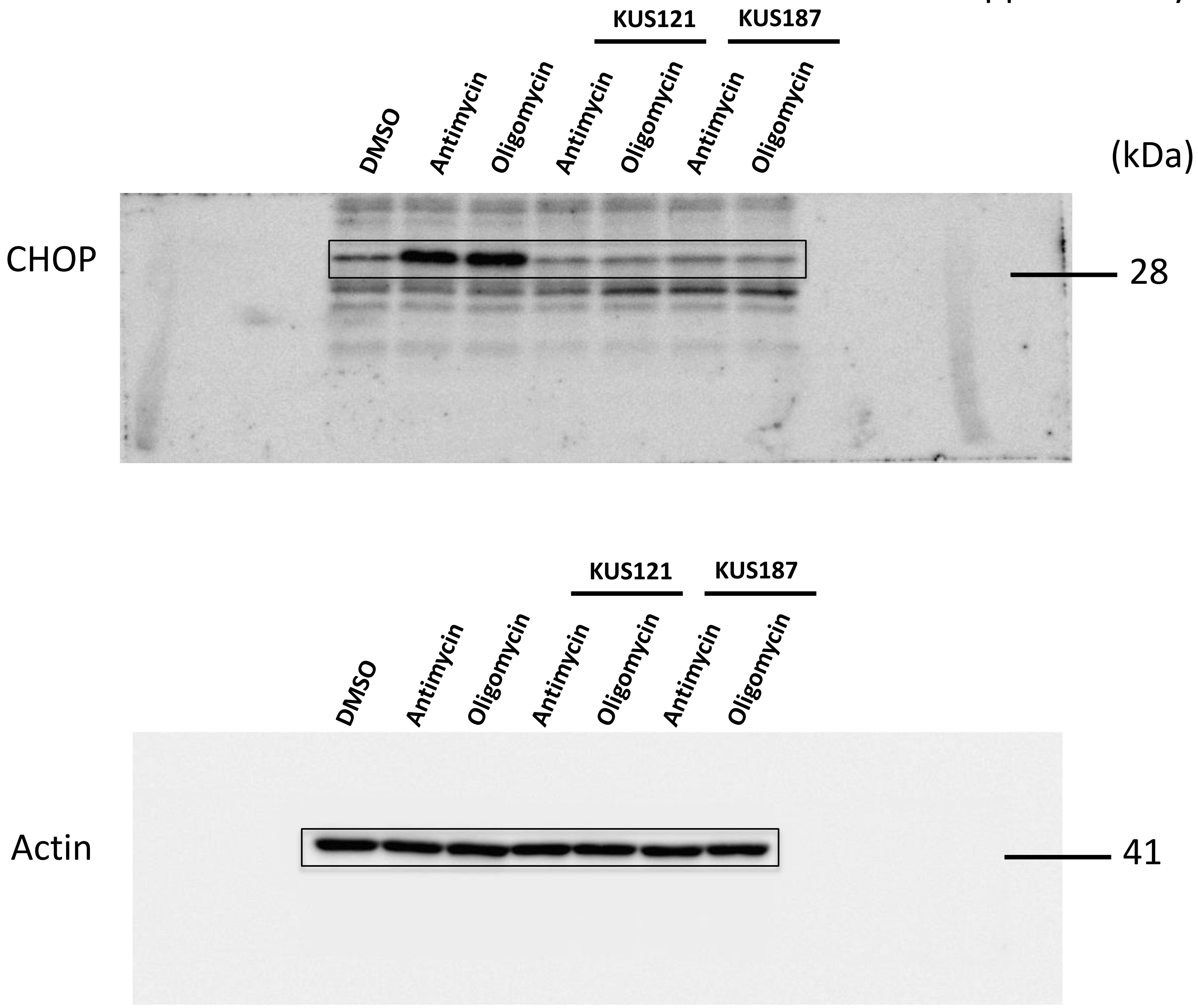

Figure 1B

Supplementary Figure S2

Grp78

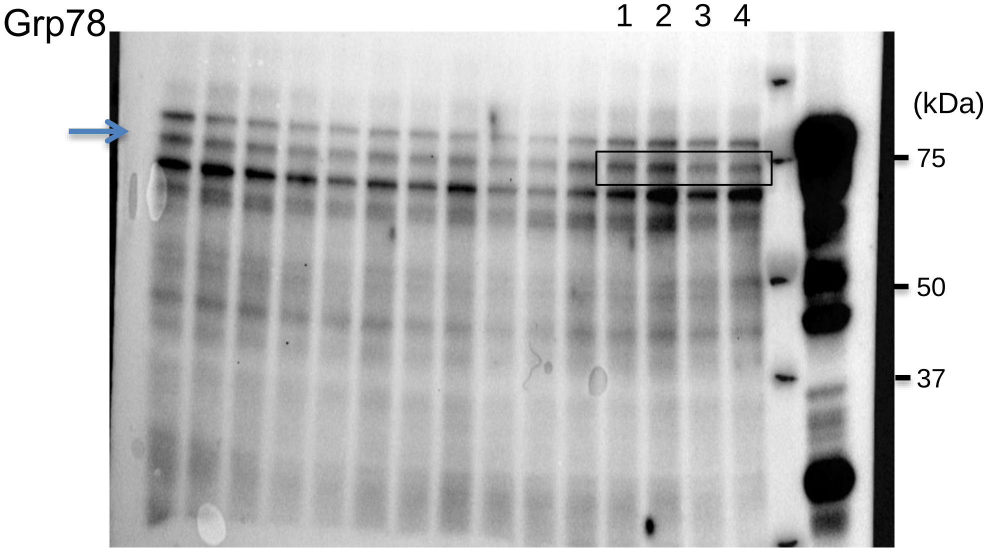

cleaved caspase3

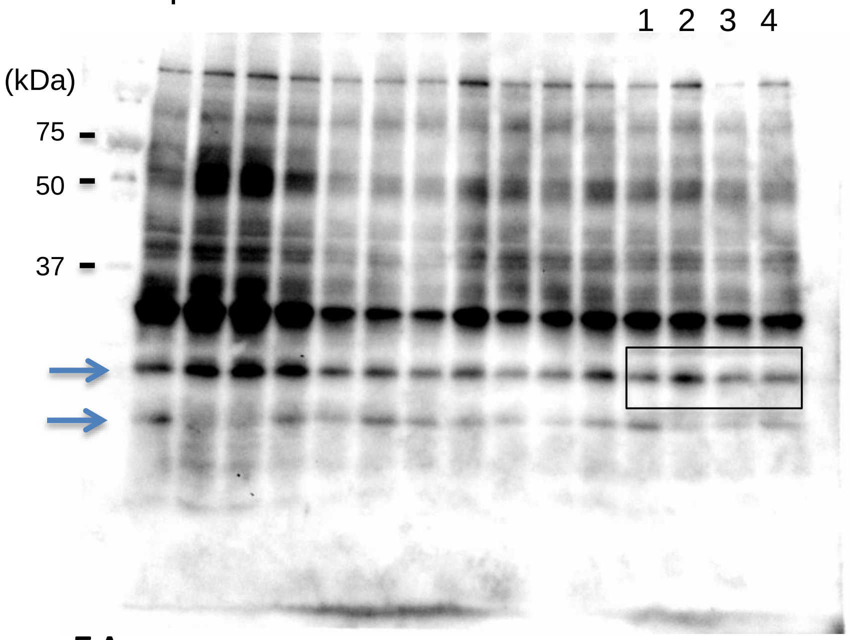

actin

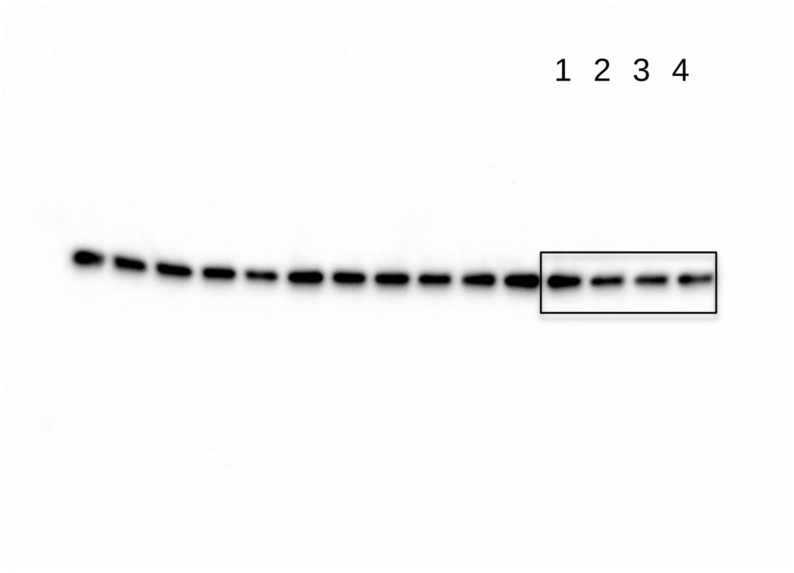

Figure 5A

Supplementary Figure S3

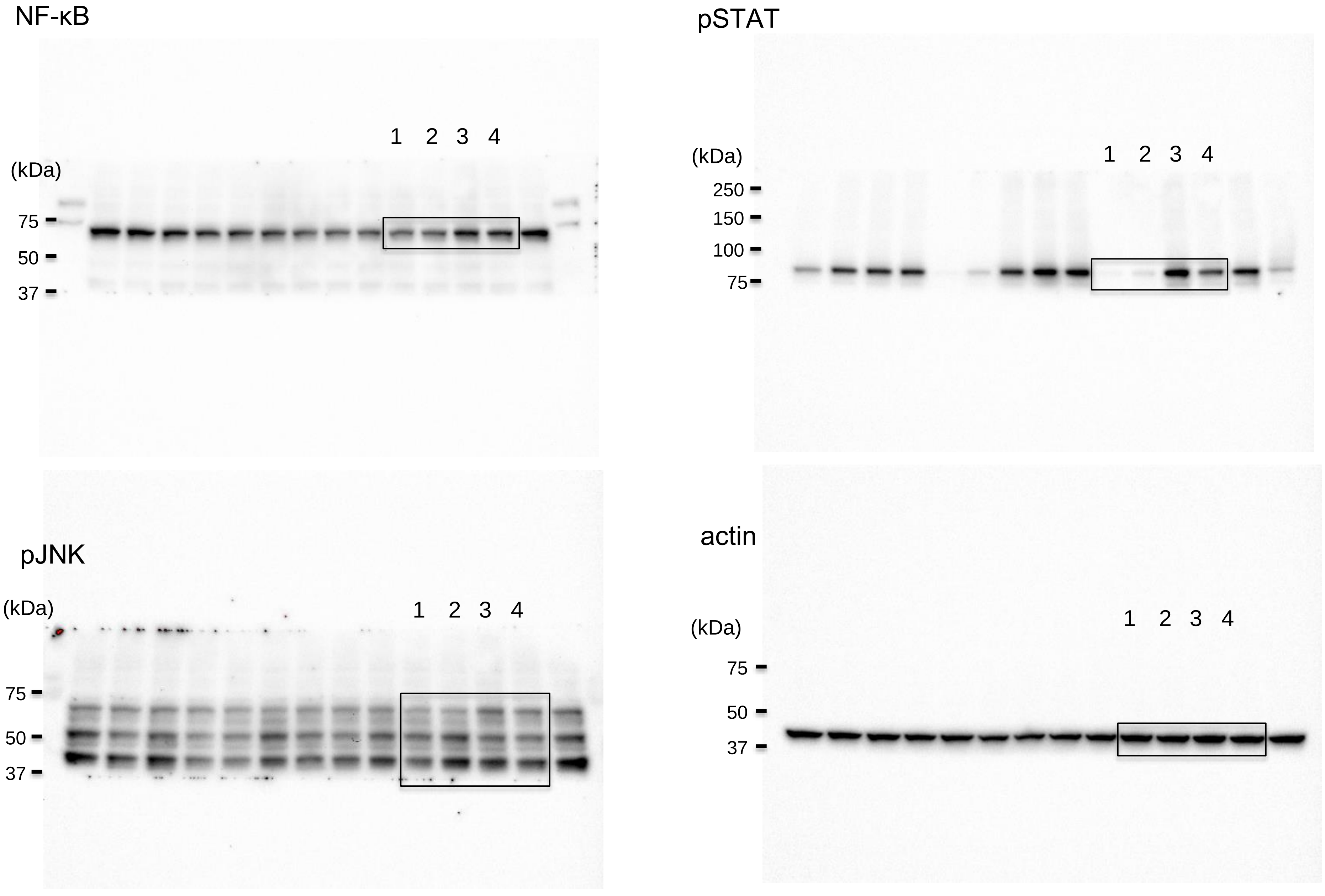

Figure 5D

Supplementary Figure Legends

Supplementary Fig S1. Complete scans of the different gels presented in the Figure 1B.

Supplementary Fig S2. Complete scans of the different gels presented in the Figure 5A. Lane 1: saline injection, lane 2-4: NMDA injection, lane 2: PBS treatment (labeled “C”), lane 3: KUS121 treatment (K121), lane 4: KUS187 treatment (K187).

Supplementary Fig S3. Complete scans of the different gels presented in the Figure 5D. Lane 1: wild type (WT), lane 2: saline injection, lane 3: PBS treatment (labeled “C”) with NMDA injection, lane 4: KUS121 treatment with NMDA injection.
